# Hydralazine-Induced Antineutrophil Cytoplasmic Antibody (ANCA)-Associated Vasculitis: A Case Report and Literature Review

**DOI:** 10.7759/cureus.24132

**Published:** 2022-04-13

**Authors:** Diala Alawneh, Amr Edrees

**Affiliations:** 1 Department of Medicine, University of Missouri Kansas City, Kansas City, USA; 2 Division of Rheumatology, University of Illinois Chicago, Chicago, USA; 3 Division of Rheumatology, Department of Medicine, University of Missouri Kansas City, Kansas City, USA

**Keywords:** anca-associated vasculitis, diffuse alveolar hemorrhage, rituximab, purpuric rash, hydralazine

## Abstract

Antineutrophil cytoplasmic antibody (ANCA)-associated vasculitis (AAV) is a disease that typically presents with multiorgan involvement. It can be idiopathic and at times drug-induced. Drugs that have been reported to cause AAV include propylthiouracil, minocycline, allopurinol, hydralazine, as in our case here, and many others. Other than stopping the offending agent, guidelines regarding treating drug-induced vasculitis (DIV) remain unclear. We present to you a case of hydralazine-induced vasculitis causing severe respiratory failure due to pulmonary hemorrhage, purpuric rash, and possible renal disease although not confirmed by biopsy. Our patient was successfully treated with rituximab and plasma exchange. This disease can be life-threatening, and aggressive treatment may be warranted at times.

## Introduction

Drug-induced vasculitis (DIV) has become more recognized throughout the years and has been associated with several medications. It usually presents as an antineutrophil cytoplasmic antibody (ANCA)-positive vasculitis and mimics idiopathic ANCA-positive vasculitis [1]. Idiopathic ANCA-positive vasculitis includes granulomatosis with polyangiitis (GPA), eosinophilic granulomatosis with polyangiitis (EGPA), and microscopic polyangiitis (MPA).

More recently, attention has been drawn to hydralazine as a cause of DIV. Hydralazine is a common direct-acting vasodilator that has been used in the treatment of hypertension and heart failure since the 1950s. It is commonly known to cause drug-induced lupus but recently has been increasingly reported to cause DIV [2]. Differentiating between both idiopathic and drug-induced AAV can be difficult. Here, we present a case of hydralazine-induced ANCA vasculitis and how to recognize it.

## Case presentation

A 71-year-old female presented with shortness of breath and a new-onset rash. She had a past medical history of hypertension and bipolar disorder, which were treated with hydralazine, carvedilol, clonidine, and lithium. Symptoms started approximately three weeks ago. She had been treated for pneumonia with levofloxacin as an outpatient with minimal improvement.

The patient was afebrile, tachypneic, and hypoxic with oxygen saturation of 81%. On examination, she was in distress with coarse breath sounds. In addition, a diffuse palpable purpuric rash was observed on her bilateral lower extremities. Initial workup showed leukocytosis, normocytic anemia, elevated creatinine at 1.35 mg/dL (baseline: 0.8 mg/dL), international normalized ratio (INR) of 1.3, prothrombin time (PT) of 15 seconds, partial thromboplastin time (PTT) of 52 seconds, erythrocyte sedimentation rate (ESR) of 81 mm/hour, C-reactive protein (CRP) of 19 mg/dL, and procalcitonin of 0.43 ng/mL (Table 1). Her arterial blood gas showed pH 7.05, pCO_2_ 65.8, pO_2_ 74.8, and HCO_2_ 17.8 on positive pressure ventilation. Chest X-ray showed diffuse bilateral opacities. She was intubated and admitted to our ICU where broad-spectrum antibiotics were started overnight.

**Table 1:** Laboratory results

Laboratory test	Patient’s result	Reference range
White cell count (WBC)	18 × 10^3^/mm^3^	4.3-10.80 × 10^3^/mm^3^
Creatinine	1.35 mg/dL	0.9-1.3 mg/dL
Erythrocyte sedimentation rate (ESR)	81 mm/hour	1-20 mm/hour
C-reactive protein (CRP)	19 mg/dL	0-1 mg/dL
Prothrombin (PT)	15 seconds	9.9-13.3 seconds
Partial thromboplastin time (PTT)	52 seconds	24-36.5 seconds
International normalized ratio (INR)	1.3	0.8-1.2
Procalcitonin	0.43 ng/mL	<0.5 ng/mL (low risk of severe infection and/or septic shock), >2 ng/mL (high risk of severe infection and/or septic shock)
pH	7.05	7.35-7.45
pCO_2_	65.8 mmHg	35-45 mmHg
pO_2_	74.8 mmHg	60-100 mmHg
HCO_2_	17.8 mEq/L	20-26 mEq/L

**Figure 1:**
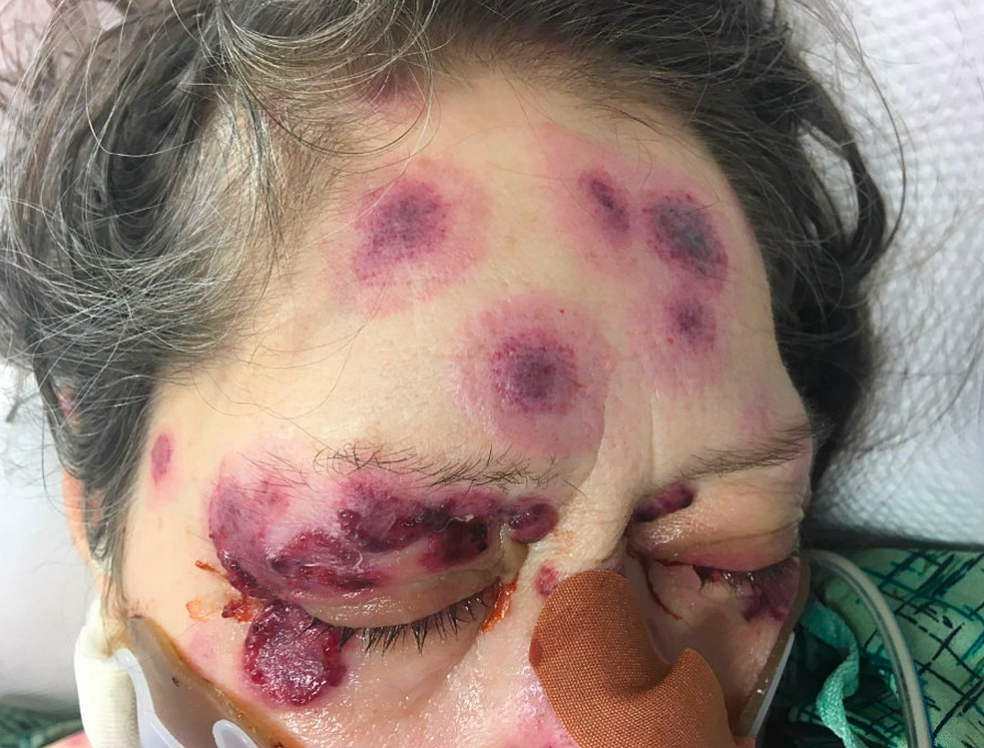
Purpuric rash involving the forehead and surrounding the eye with multiple lesions on the eyelid

**Figure 2:**
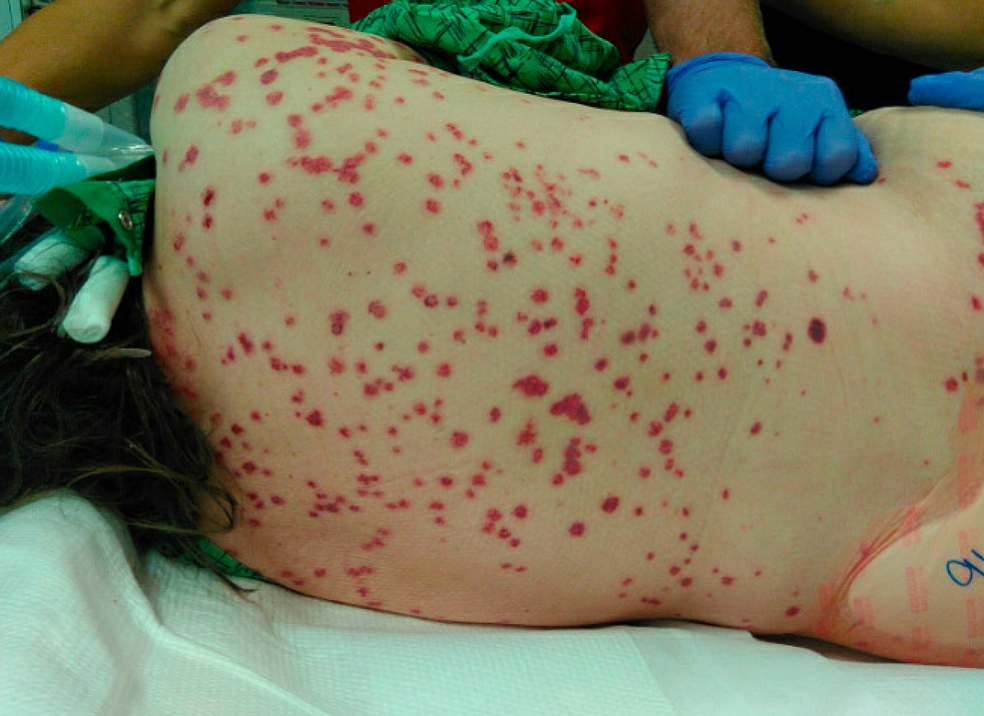
Diffuse purpuric rash on the patient’s back

**Figure 3:**
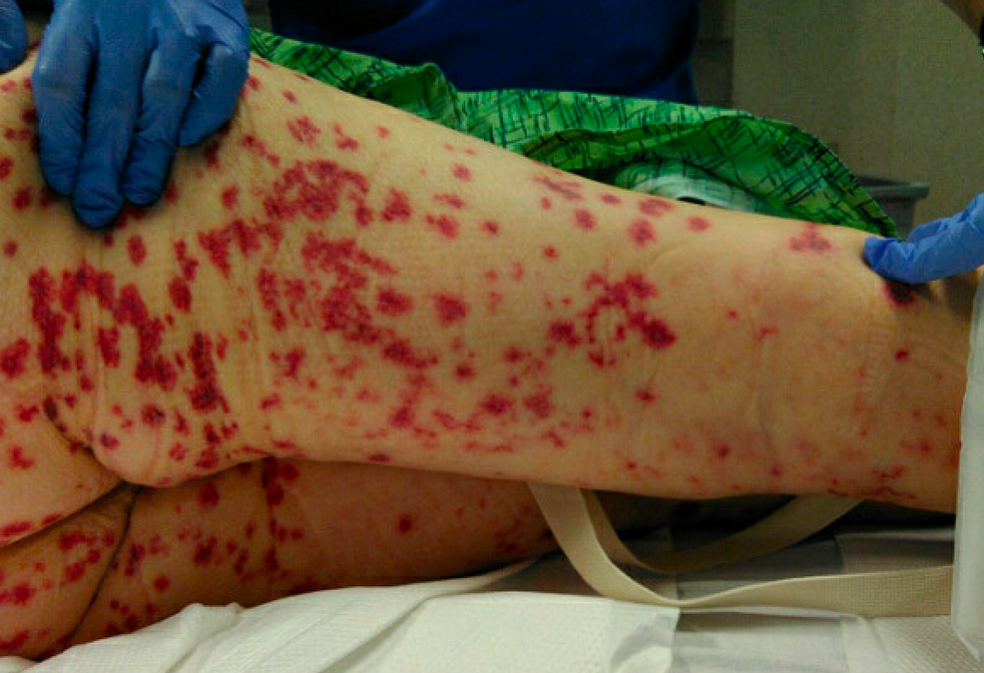
Diffuse purpuric rash on the patient’s lower extremities and buttocks

**Figure 4:**
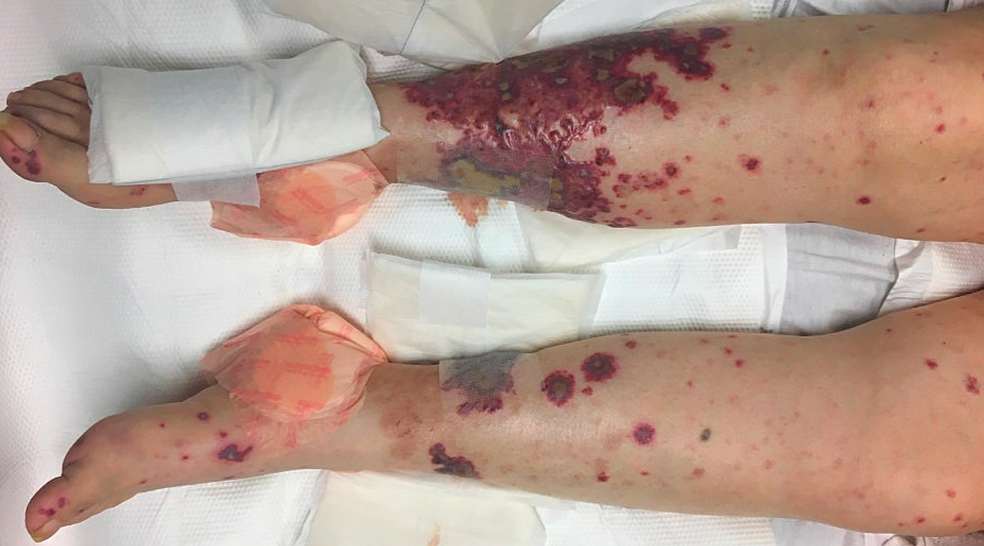
Purpuric rash on the patient’s lower extremities

On day 2, a chest CT was done showing diffuse ground-glass opacities in all five lobes concerning pulmonary hemorrhage versus infection. By then, her rash had progressively become worse and was now involving her upper extremities and face (Figures 1-4). Rheumatology was consulted. Initial serologies, including antinuclear antibody (ANA), anti-double-stranded DNA, anti-Smith, antinuclear ribonucleoprotein (anti-RNP), Scl-70, and anti-Sjögren’s syndrome antigen A (SSA) and B (SSB) antibodies, were all negative. Her C3 and C4 complement levels were within normal limits. Urine analysis showed numerous red blood cells (RBCs). The urine protein/creatinine ratio was 1.4. She underwent bronchoscopy, and the initial results showed an RBC count of 761/UL and a total neutrophil count of 281/UL. Due to concern for a small-vessel vasculitis, our patient was started on methylprednisolone 1 g IV twice daily for three days. A more frequent steroid dosing regimen was used due to the very rapid progress of the lesions on the patient’s face and eyes and the fear that it would threaten her vision.

Further workup revealed positive perinuclear antineutrophil cytoplasmic antibody (P-ANCA) with positive myeloperoxidase (MPO) but otherwise negative proteinase 3 (PR3) antibody, glomerular basement membrane antibody, cryoglobulin, serum protein electrophoresis, HIV, and hepatitis serologies. Skin biopsy showed leukocytoclastic (necrotizing) vasculitis. She was diagnosed with ANCA-positive vasculitis. This was thought to be due to her hydralazine. In addition to stopping the medication, due to the severity of her disease, the patient was started on rituximab 375 mg/m^2^ weekly for four weeks per the Rituximab in ANCA-Associated Vasculitis (RAVE) protocol and plasma exchange in addition to methylprednisolone taper.

The patient’s clinical status improved gradually during her stay. On day 10, she was extubated successfully. She completed rituximab and seven sessions of plasma exchange before discharge on prednisone taper to follow up as an outpatient.

## Discussion

There are many theories regarding the mechanism of action of hydralazine in inducing vasculitis. One theory is that hydralazine binds to myeloperoxidase (MPO) in the neutrophils, leading to apoptosis and the release of cytotoxic products [1]. Another theory is that it decreases DNA methyltransferase expression, which affects the suppression of MPO and proteinase 3 (PR3) [3].

The incidence of DIV itself remains unknown [4]. However, the incidence of hydralazine-induced vasculitis, which is thought to be dose-dependent, has been reported to be 5.4% and 10.4% at drug doses of 100 mg and 200 mg daily for more than three years, respectively [1,3]. Female gender, those with thyroid disease, and those taking high doses of hydralazine are considered risk factors for developing DIV [4]. Our patient, despite the unknown duration of hydralazine use, was taking 100 mg three times a day and is a female, which put her at a higher risk.

Hydralazine-induced vasculitis has been known to most commonly involve the kidneys and skin and less frequently the lungs, joints, and neurons [1]. Pulmonary-renal syndrome, although rare, has been reported as well [3,4]. In our patient, despite not having a renal biopsy to confirm renal involvement due to the presence of RBCs in the urine, elevated creatinine, and proteinuria, we believe it was most likely a pulmonary-renal syndrome picture, just not as severe. When comparing entities caused by hydralazine, it was found that patients with hydralazine-induced vasculitis had a more severe course than hydralazine-induced lupus. This is thought to be due to renal vasculitis [5].

Diagnosing hydralazine-induced vasculitis and differentiating it from idiopathic vasculitis can be tricky. The presence of symptoms with a history of drug use, duration of therapy, positive serology, and resolution of symptoms with stopping medication are used to establish the diagnosis [4]. Typically, idiopathic vasculitis presents with ANCA positive to one neutrophilic antigen. However, in addition to high MPO-ANCA titers, drug-induced vasculitis serology can reveal antinuclear antibodies (ANA), anti-DNA, anti-histone antibodies, anti-Sjogren’s syndrome A, antiphospholipid antibodies, and atypical ANCAs [1,2,4]. In addition, it is thought that more immune deposits may actually be seen compared to idiopathic vasculitis pauci immune vasculitis on biopsy of the affected organ [1].

Withdrawal of hydralazine is the first step in the treatment of hydralazine-induced vasculitis, especially in mild cases. Pulse dose steroids are generally required for active organ involvement [6]. More severe cases, including those with renal involvement, require immunosuppressant agents. The case review of Yokogawa and Vivino of 68 cases of hydralazine-induced vasculitis showed 48 patients requiring immunosuppressive therapy. A total of 55 patients had renal involvement [1]. Cyclophosphamide is considered the first-line therapy. Rituximab has been reported to be effective in management as well [7]. The exact duration of immunosuppressive therapy remains unknown. However, maintenance therapy generally is not needed once the offending drug has been stopped as patients usually do not have relapses [6].

## Conclusions

Hydralazine-induced AAV can be a severe life-threatening disease. A high index of suspicion is required for early diagnosis and discontinuation of hydralazine. 

The clinical spectrum of hydralazine-induced AAV can involve the lungs, kidneys, and skin. Severe cutaneous vasculitis and alveolar hemorrhage occurred in our patient.

Currently, there are no guidelines for the use of immunosuppressive medications in the treatment of hydralazine AAV. The use of rituximab in our patient was effective and well-tolerated. Lack of recurrence after the withdrawal of the offending drug can differentiate hydralazine-induced ANCA vasculitis from the classic ANCA vasculitis and eliminate the need for long-term maintenance immunosuppressive medications. We hope that more published data regarding this subject will help guide physicians on how best to treat similar cases.
